# Adjuvant chemoradiotherapy for locoregionally advanced and high-risk salivary gland malignancies

**DOI:** 10.1186/1758-3284-3-31

**Published:** 2011-07-26

**Authors:** Aaron W Pederson, Joseph K Salama, Daniel J Haraf, Mary Ellen Witt, Kerstin M Stenson, Louis Portugal, Tanguy Seiwert, Victoria M Villaflor, Ezra EW Cohen, Everett E Vokes, Elizabeth A Blair

**Affiliations:** 1 Department of Radiation Oncology, Memorial University Medical Center, 4700 Waters Avenue, Savannah, GA 31404, USA; 2 Department of Radiation Oncology, Duke University School of Medicine, Box 3085 Durham, NC, 27710, USA; 3 Department of Radiation and Cellular Oncology, University of Chicago, 5758 S. Maryland Ave, MC 9006, Chicago, IL 606037, USA; 4 Comprehensive Cancer Center, University of Chicago, 5841 S Maryland Ave, Chicago, IL, USA; 5 Section of Otolaryngology/Head and Neck Surgery, Department of Surgery, University of Chicago 5841 S. Maryland Ave, MC 1035, Chicago, IL, USA; 6 Section of Hematology/Oncology, Department of Medicine University of Chicago, 5841 S. Maryland Ave, MC 2115, Chicago, IL, 60637, USA

## Abstract

**Background:**

To report the outcomes of patients with locoregionally advanced and high- risk salivary gland malignancies treated with surgery followed by adjuvant chemoradiotherapy.

**Methods:**

From 09/1991 - 06/2007, 24 high-risk salivary gland cancer patients were treated with surgery, followed by adjuvant chemoradiotherapy for high-risk pathologic features including, perineural involvement, nodal involvement, positive margins, or T3/T4 tumors. Chemoradiotherapy was delivered for 4-6 alternating week cycles: the most common regimen, TFHX, consisted of 5 days paclitaxel (100 mg/m^2 ^on d1), infusional 5-fluorouracil (600 mg/m^2^/d × 5d), hydroxyurea (500 mg PO BID), and 1.5 Gy twice daily irradiation followed by a 9-day break without treatment.

**Results:**

Median follow-up was 42 months. The parotid gland was more frequently involved (n = 17) than minor (n = 4) or submandibular (n = 3) glands. The median radiation dose was 65 Gy (range 55-68 Gy). Acute treatment related toxicity included 46% grade 3 mucositis and 33% grade 3 hematologic toxicity. Six patients required feeding tubes during treatment. One patient progressed locally, 8 patients progressed distantly, and none progressed regionally. Five-year locoregional progression free survival was 96%. The 3 and 5 year overall survival was 79% and 59%, respectively. Long-term complications included persistent xerostomia (n = 5), esophageal stricture requiring dilatation (n = 1), and tempromandibular joint syndrome (n = 1).

**Conclusions:**

Surgical resection followed by adjuvant chemoradiotherapy results in promising locoregional control for high-risk salivary malignancy patients.

## Background

Salivary gland cancers comprise 3-6% of all head and neck malignancies [1,2]. Although they are commonly grouped together, these cancers are histologically diverse with varying natural histories. Surgery is the established initial treatment for salivary gland malignancies. Adjuvant radiotherapy in patients with high-grade tumors or high- risk features, improves locoregional control in select patients undergoing surgical resection compared to surgical resection alone [1,3-9]. Chemotherapy is not routinely used outside of the palliative or recurrent disease setting for salivary gland tumors. The most commonly used regimen is cyclophosphamide, doxorubicin, and cisplatin (CAP) with response rates of 27-83%, and few of long duration [10-13]. In small, single-institution series, survival rates of 60-72% at 5 years have been reported in patients receiving chemoradiotherapy for salivary malignancies [14-18]. Previous studies from our institution have demonstrated the combination of 5-FU, hydroxyurea, and radiotherapy (FHX) an effective regimen for head and neck cancer (particularly those of squamous histology)[19]. Based on these favorable results we sought to improve locoregional control in patients with advanced and high- risk salivary gland malignancies, utilizing FHX based regimens. The present study was undertaken to report the outcomes of patients treated adjuvantly with concurrent FHX based chemoradiotherapy for salivary glands carcinomas.

## Methods

### Patient population

Between 09/1991 and 06/2007, 72 consecutive patients with primary or recurrent salivary gland carcinoma underwent evaluation and treatment with radiation therapy with or without surgery or chemotherapy at University of Chicago Hospitals or at a participating hospital on a University of Chicago protocol. From this cohort, 14 patients with distant metastases, 10 patients with prior radiotherapy or recurrent disease, and 15 patients receiving only radiotherapy as adjuvant treatment were excluded from this analysis. An additional 9 patients treated without surgery were excluded. The 24 remaining patients who received surgery followed by adjuvant chemoradiotherapy formed our study population. All patients were evaluated before therapeutic intervention by a medical oncologist, radiation oncologist, and head and neck surgeon. Initial staging procedures consisted of a history and physical, complete head and neck exam, biopsy and/or tumor resection if feasible, tumor mapping, dental evaluation, head, neck and chest computed tomography (CT) scan, as well as quality of life, and speech and swallowing assessment in most patients. All patients signed informed consent before beginning therapy. The Institutional Review Board at the University of Chicago approved this study.

### Treatment

All patients underwent macroscopically complete oncologic surgical resection. The type and extent of surgery was dependent on the primary site, surgeon's clinical judgment, and the patient's willingness to undergo resection. In general, surgical resection of all known disease at the primary and neck was recommended for all patients. The primary goal of surgical resection was to preserve cosmetic and functional outcome without sacrificing locoregional control. Neck dissection was performed most often with therapeutic intent, but also less commonly for diagnostic purposes.

Adjuvant chemoradiotherapy was recommended for high-risk pathologic features such as perineural involvement, nodal involvement, or involved surgical margins. Patients received 4-6 alternating week cycles of FHX-based chemoradiotherapy based on pathologic risk factors and radiation fractionation. Each 14-day cycle consisted of five days of concurrent chemoradiotherapy followed by nine days without chemotherapy or radiotherapy. Some patients were treated on protocols investigating the integration of new agents with FHX. These included irinotecan (5-15 mg/m^2^/d × 5d), combined with concurrent infusional 5-fluorouracil (600 mg/m^2^/d × 5d), and hydroxyurea (500 mg PO BID); gemcitabine (50-300 mg/m^2 ^on d1), combined with concurrent paclitaxel (100 mg/m^2 ^on d1) and infusional 5-fluorouracil (600 mg/m^2^/d × 5d), as well as bevacizumab (2.5-10 mg/kg/m^2 ^on d1) combined with infusional 5-fluorouracil (600-800 mg/m^2^/d × 5d) and hydroxyurea (500-1000 mg PO BID) (BFHX). Patients treated off protocol were commonly treated with 5 days of concurrent paclitaxel (100 mg/m^2 ^on d1), infusional 5-fluorouracil (600 mg/m^2^/d × 5d), and hydroxyurea (500 mg PO BID) (TFHX).

All patients treated after 1997 underwent CT based treatment planning. All were immobilized in custom masks. Treatment volumes were individualized based on extent of surgical resection, high-risk pathological features and extent of tumor invasion. Initially 3D conformal radiotherapy was used, and as it became increasingly available, intensity-modulated radiotherapy was used. All patients were treated on 6 MV linear accelerators. Most patients were treated with 1.5 Gy twice daily with a minimum six-hour interfraction interval. Tumor in close proximity to critical neural structures received a single daily fraction of 2 Gy. Radiotherapy dose was individualized based on adverse pathologic features. In general, 60 Gy was prescribed for perineural invasion or > 1 lymph node with metastatic spread, or T3/T4 tumor status. Patients with positive margins and extra nodal extension were prescribed 66 Gy, and those with gross residual disease were prescribed 70-75 Gy.

All patients were evaluated weekly during chemoradiotherapy for adverse events. Acute toxicity was graded based on the CTCAE v 3.0. Following completion of chemoradiotherapy patients underwent contrast enhanced CT neck and chest one month following the completion of therapy, then every three months for 2 years then every 6 months until five years following completion of treatment. Late toxicity was graded using the RTOG late toxicity scoring system.

### Statistical analysis

Three- and 5-year estimates of the probability of locoregional control, distant-metastases free survival, disease-free survival, and overall survival were calculated using the Kaplan-Meier method [20]. Clinical and disease variables considered as potential predictors of outcome on univariate analysis were: primary site, histology, T-stage, lymph node metastasis, radiation dose, radiation fractionation, and chemotherapy regimen. Events were measured from the date of initial surgery, with patient follow-up reported to the date last seen in clinic or identified through the Social Security Death Index.

## Results

### Patient Characteristics

Baseline characteristics of the population are listed in Table 1. The median age was 62 years (range, 26-79 years). Sixteen patients (63%) were male. The parotid gland was more frequently involved (n = 17) than minor (n = 4) or submandibular (n = 3) gland. Tumor histology was as follows: 5 adenoid cystic (21%), 5 mucoepidermoid (21%), 3 adenocarcinoma (13%), 3 squamous cell carcinoma (13%), 3 poorly differentiated (13%) and 6 other (25%). All primary tumors were retrospectively staged in accordance with the 2002 American Joint Committee on Cancer (AJCC) staging system. The three patients with squamous cell carcinoma had no history of skin cancers and full dermatologic examination demonstrated no evidence of cutaneous lesions. There was agreement that the tumors arose from salivary gland tissue and did not represent metastatic disease from another primary tumor.

**Table 1:** Baseline patient characteristics

Patient Characteristic	Number of Patients (%)
Male:Female Ratio	16:8

Average Age (range) years	62 (26-79)

Primary Site	
Parotid	17 (71%)
Submandibular	3 (13%)
Minor - Base of Tongue	2 (8%)
Minor - Sphenoid Sinus	1 (4%)
Minor - Ethmoid Sinus	1 (4%)
Histology	
Adenoid Cystic Carcinoma	5 (21%)
Mucoepidermoid Carcinoma	5 (21%)
Adenocarcinoma	3 (13%)
Squamous Cell Carcinoma	3 (13%)
Poorly Differentiated Carcinoma	3 (13%)
Carcinoma Ex-pleomorphic Adenoma	2 (8%)
Myoepithelial Carcinoma	1 (4%)
Salivary Duct Carcinoma	1 (4%)
Anaplastic Carcinoma	1 (4%)

T-Stage	
1	1 (4%)
2	8 (33%)
3	6 (25%)
4a	7 (29%)
4b	2 (8%)

N-Stage	
0	6 (25%)
1	3 (13%)
2a	0
2b	14 (58%)
2c	1 (4%)
3	0

Stage	
II	1 (4%)
III	2 (8%)
IVA	19 (79%)
IVB	2 (8%)

As shown in Table 1, the distribution of clinical or pathologic T-stage was: 1 T1, 8 T2, 6 T3, 7 T4A, and 2 T4B. The distribution of clinical or pathologic N-stage was: 6 N0, 3 N1, 0 N2A, 14 N2b, 1 N2c, and 0 N3. Overall stage breakdown was as follows: 1 stage II, 2 stage III, 19 stage IVA, and 2 stage IVB. Three patients received induction therapy before concurrent chemoradiotherapy. Fifteen patients (63%) presented with palpable or radiographic evidence of N2b-N3 cervical lymphadenopathy and 21 underwent neck dissection at some point during treatment. The median radiation dose was 65 Gy (R 55 Gy - 68 Gy). Median patient follow-up was 42 months (range, 7-121 months) for all patients and 43 months for survivors (range 7-95 months).

### Patterns of Recurrence

**Figure 1:**
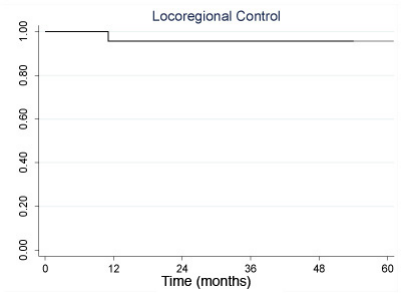
**Kaplan-Meier analysis of locoregional control**.

Locoregional recurrence was uncommon. No patient recurred regionally. One patient recurred locally at 14 months. This patient with a T4aN2b high-grade mucoepidermoid carcinoma of the base of tongue underwent surgical salvage and lived another 9 years before dying of a primary lung cancer. Locoregional progression free survival at 3 and 5 years was 96% (95% CI: 73, 99) as shown in Figure 1. Eight patients progressed distantly. The lung was the most common site involved (6 patients), followed by brain (2 patients) and liver (2 patients). The mean time to distant progression was 23 months (range, 4 - 75 months). Two patients received palliative chemotherapy and 1 patient received whole brain radiotherapy for brain metastases. The mean time to death following distant progression was 19 months (range 1-38 months). No patient receiving postoperative chemotherapy prior to chemoradiotherapy progressed distantly. Two patients each with adenoid cystic and poorly differentiated histology developed lung metastases.

### Disease-Free and Overall Survival

**Figure 2:**
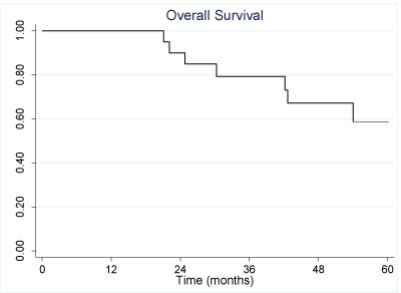
**Kaplan-Meier analysis of overall survival**.

The 3 and 5 year overall survival was 79% (95% CI: 54, 92) and 59% (95% CI: 31, 79), respectively, as shown in Figure 2. Median and average survival were 42 months and 52 months, respectively. The 3 and 5 year disease free survival was 62% (95% CI: 38, 79) and 55% (95% CI: 31, 74), respectively. Table 2 summarizes disease-free survival according to clinical and disease characteristics. Univariate analysis of the clinical and pathological variables assessed did not reveal any differences in disease-free survival. Three-year disease free survival in patients with N2 disease was 49% compared to 86% in those with N0 or N1 status (p = 0.14). There was also a trend to improved disease free survival in patients receiving a taxane-containing chemotherapy (80% vs. 45%, p = 0.17). Univariate analysis for overall survival demonstrated that only nodal status was associated with statistically significant improvements: N2 patients had a 5-year survival rate of 42% vs. 100% for patients with N0 or N1 disease (p = 0.03). No patients with squamous cell carcinoma experienced cancer recurrence: 2 patients are alive at 39 and 63 months, while a third patient died of natural causes at 98 months.

**Table 2:** Disease-free survival by clinical and disease characteristics

Factor	Progressors/Total	3-y DFS (%)	p-value
T-stage			0.99
T1-2	6/14	69	
T3-4	5/10	56	

Lymph Node Mets			0.14
N0/N1	2/8	86	
N2	9/16	49	

Tumor Grade			0.61
Low	3/5	53	
High	8/19	64	

Perineural Inv.			0.29
Absent	4/11	70	
Present	7/13	55	

Age			0.86
< 55	4/9	50	
> 55	7/15	71	

Gender			0.28
Male	6/16	72	
Female	5/8	44	

Dose > 63 Gy			0.81
Yes	6/15	63	
No	5/9	50	

TFHX			0.17
Yes	3/12	80	
No	8/12	45	

### Toxicity

Acute treatment related toxicity included 46% grade 3 mucositis and 33% grade 3 hematologic toxicity. No grade 4 toxicity was recorded during therapy. Six patients required feeding tubes during treatment. Long-term complications included persistent xerostomia in 5 patients, esophageal stricture requiring dilatation in 1 patient, and tempromandibular joint (TMJ) syndrome in 1 patient. Three patients (13%) had feeding tubes at last follow-up or death. No patients required tracheostomy during or after therapy.

## Discussion

Concurrent chemotherapy and radiotherapy has been shown to improve locoregional control and survival for many tumor types [21]. For head and neck cancers, primarily of squamous origin, concurrent chemoradiotherapy has been shown to result in an absolute 10% 5 year overall survival benefit [22]. Concurrent chemoradiotherapy has been established as the standard of care for many patients either definitively or adjuvantly with locally advanced head and neck cancer.

Despite the proven benefit of concomitant chemoradiotherapy for squamous and poorly differentiated head and neck cancers, the impact for locoregionally advanced and salivary gland malignancies is much less clear. While several case reports of sequential or concurrent chemoradiotherapy for locoregionally advanced salivary tumors exist, there is a paucity of data describing chemoradiotherapy in large series. Airoldi et al described 6 patients with inoperable salivary malignancy treated with radiotherapy to 66 Gy with concurrent cisplatin followed by 3 cycles of cisplatin and etoposide. Three patients had a complete response to therapy and median survival was 18 months [23]. In the largest previously reported series we are aware of, seventeen patients with Stage III or IV salivary malignancies were treated with definitive chemoradiotherapy consisting of daily radiotherapy to ~66 Gy and 2 monthly cycles of cyclophosphamide, cisplatin, and pirarubicin [17]. Nine patients had surgery for residual disease and 4 (24%) patients had pathologic complete response with chemoradiotherapy alone. The current analysis describes the largest series of patients treated with concurrent chemoradiotherapy for advanced, high- risk salivary gland malignancies. Our results compare favorably with the smaller previously described series of chemoradiotherapy, as the median survival for patients was 42 months with one local failure noted.

The locoregional control rate in this report (96%) compares favorably to large series of patients treated with definitive surgery and radiotherapy or radiotherapy alone. Published reports of surgery with or without adjuvant radiotherapy for salivary cancer demonstrate generally mediocre locoregional control rates, especially for Stage IV disease. The University of Florida reported locoregional control rates of 66% and 81%, for T4 tumors treated with surgery and surgery plus radiotherapy, respectively. Chen et al has reported on 45 patients with node-negative salivary malignancies treated with definitive radiotherapy to ~66 Gy. The ultimate local control rate was 57% and significantly worse for T3-4 tumors [24]. Laramore has summarized the published literature of definitive photon/electron radiotherapy for salivary gland malignancy and found a local control rate of only 30%. This is in contrast to the rate found for definitive fast neutron therapy, with a local control rate of 67% [25]. In a matched-pair analysis of surgery versus surgery plus radiotherapy, Armstrong et al reported that local control for stage III and IV disease was only 51.3% for combined treatment versus 16.8% for surgery alone [1]. In a series of over 500 salivary malignancies, postoperative radiotherapy improved local control over surgery alone for T3-4 tumors (84% vs. 18%), in close (95% vs. 55%) and incomplete resection (82% vs. 44%), and presence of perineural invasion (88% vs. 60%) [9]. Data from the Dutch Head and Neck Oncology Cooperative Group demonstrated that postoperative radiotherapy significantly improved regional control in the node positive neck, 86% vs. 62% for surgery alone. There were zero regional failures in the 18 node positive patients treated with chemoradiotherapy in this series. Current NCCN recommendations for adjuvant radiotherapy include intermediate and high grade, adenoid cystic histology, close or positive margins, perineural invasion, lymph node metastases and lymphovascular invasion.

Additionally, our results are promising when compared to patients treated with neutron therapy. In the University of Washington series of 279 patients, the 6-year locoregional control was 59% and late Grade 3-4 toxicity was 10% [26]. In subset analysis, stage III-IV (locoregional control 49%) and definitive radiotherapy (locoregional control 40%) were associated with worse outcomes. While the Medical Research Council (MRC) trial demonstrated improved locoregional control for patients with unresectable salivary gland malignancies treated with fast neutron therapy, severe and life-threatening complication rates were 69% and 15%, respectively after neutron radiotherapy versus 33% and 8% after photon radiotherapy [2]. The toxicity of neutron therapy and limited accessibility, only 3 operating neutron therapy centers in the United States, make photon therapy a more reasonable and widely available treatment. The acute and chronic toxicity of our regimen was encouraging with limited acute mucositis and hematologic toxicity. Chronic complications were also limited given the advanced stage of the treated patients. No fatalities in this series were thought to be treatment-related.

The dominant pattern of progression seen in our series was distant metastases, a relationship that has been noted in previous studies of FHX-based chemoradiotherapy [19]. This pattern of progression is consistent with the natural history of salivary gland malignancy. One hypothesis is that reducing distant progression requires improved systemic therapy. There are a number of Phase I and II trials that have investigated systemic therapy with mixed results [26]. Molecular targeted agents, including lapatinib and trastuzumab, have been used as monotherapy in the metastatic setting with generally cytostatic results [27-29]. Future trials of chemoradiotherapy plus molecular targeted therapy may be warranted for high-risk disease in the adjuvant setting and is warranted in the setting of locally advanced disease.

In patients with high- risk salivary gland malignancies treated with adjuvant chemoradiotherapy, locoregional control rates were favorable compared to other published reports in the literature. In particular, locoregional control was high even in node positive patients (93%) compared to 86% as reported in post-operative patients from The Dutch Head and Neck Oncology Cooperative Group [9]. Our data are limited by the retrospective nature of the study, but the same is true of almost all other published salivary gland malignancy reports. Unlike squamous cell carcinomas of the head and neck, where chemoradiotherapy has emerged as the dominant treatment paradigm, little evidence supports its use for salivary gland malignancy. However, our locoregional control rates suggest that chemotherapy acts as a radiosensitizing agent. The rare and heterogenous nature of salivary gland cancer limits the feasibility of prospective clinical trials with adequate power. Currently, the RTOG has initiated a study to investigate the role of adjuvant cisplatin based chemoradiotherapy for high-risk salivary gland malignancies.

In conclusion surgical resection followed by adjuvant chemoradiotherapy was associated with promising outcomes in this series. Ongoing investigations will be needed to verify the role of adjuvant chemoradiotherapy. Integration of active systemic agents are needed to reduce distant metastatic progression.

## Conflict of interests statement

The authors declare that they have no competing interests.

## Authors' contributions

All authors read and approved the final manuscript. AP was responsible for conception, acquisition of data, interpretation of data, manuscript writing, review of final manuscript. JS, DH, and EAB were responsible for treating subjects, conception, acquisition of data, interpretation of data, manuscript writing, and review of final manuscript. MW was responsible for acquisition of data and review of final manuscript. KS, LP, TS, VV, EC, and EV were responsible for treatment of subjects and review of final manuscript.
